# Assessing the impact of radiofrequency ablation on hilar cholangiocarcinoma: a systematic review and meta-analysis

**DOI:** 10.1097/JS9.0000000000003242

**Published:** 2025-09-04

**Authors:** Haibin Zhou, Hayat Khizar, Jing Wang, Jianfeng Yang

**Affiliations:** a Department of Gastroenterology, Affiliated Hangzhou First People’s Hospital, School of Medicine, Westlake University, Hangzhou, China; b Department of Surgery, The Fourth Affiliated Hospital of School of Medicine, and International School of Medicine, International Institutes of Medicine, Zhejiang University, Yiwu, China; c Department of Gastroenterology, Hangzhou Cancer Hospital, Hangzhou, China; d Key Laboratory of Integrated Traditional Chinese and Western Medicine for Biliary and Pancreatic Diseases of Zhejiang Province, Hangzhou, China; e Department of Gastroenterology, Hangzhou Institute of Digestive Diseases, Hangzhou, China

**Keywords:** endobiliary stents, hilar cholangiocarcinoma, meta-analysis, palliative therapy, radiofrequency ablation

## Abstract

**Background and aims:**

Hilar cholangiocarcinoma (HC) is a challenging malignancy with limited treatment options. Radiofrequency ablation (RFA) has emerged as a potential palliative treatment, but its efficacy and safety remain controversial. This systematic review and meta-analysis aimed to evaluate the impact of RFA + stent (RFA + S) and stent only (S-only) on HC patients.

**Methods:**

We conducted a systematic search of PubMed, Embase, and Scopus databases for studies published up to December 2024. Eligible studies reporting RFA outcomes were included. The primary outcome was overall survival (OS). Secondary outcomes included stent patency (SP), stent dysfunction, and adverse events. We calculated hazard ratios (HR) and odds ratios (OR) using random-effects models.

**Results:**

This analysis included eleven studies (*n* = 874 patients) that met the inclusion criteria. The pooled HR for OS was 0.74 (95% confidence interval [CI]: 0.61–0.89, *P* = 0.002), and SP was 0.77 (95% CI: 0.61–0.97; *P* = 0.03), showing a significant difference in favor of the RFA group. The OR of overall adverse events was 1.48 (95% CI: 0.59–3.72), cholangitis was 1.71 (95% CI: 0.78–3.75), pancreatitis was 1.03 (95% CI: 0.19–5.50), and liver abscess was 0.62 (95% CI: 0.07–5.24), showing no difference (*P* > 0.05) between the two groups. Patients with chemotherapy showed better survival, indicated by an HR of 0.57 (95% CI: 0.40–0.81). Subgroup analyses also showed that randomized controlled trial (RCT) studies had no difference from non-RCT studies.

**Conclusions:**

This meta-analysis suggests that the application of RFA offers better survival and stent patency benefits with comparable adverse events.

## Introduction

Hilar cholangiocarcinoma (HC), or Klatskin tumor, is a rare and aggressive malignancy that originates from the biliary epithelium at the junction of the right and left hepatic ducts. It comprises about 60%–70% of all cholangiocarcinoma and is associated with a poor prognosis^[1]^. The 5-year survival rates of HC seldom surpass 10% of patients. The poor results seen in patients with HC can be related to the challenging anatomical position, the potential for early local invasion, and common presentation at advanced stages^[2]^.

**HIGHLIGHTS**

- In hilar cholangiocarcinoma patients, radiofrequency ablation (RFA) plus stenting improved overall survival (hazard ratios [HR] = 0.74) and stent patency (HR = 0.77) compared to stenting alone.
- Safety was similar across the RFA + stent and stent-only groups for adverse events like cholangitis, pancreatitis, and liver abscesses.
- Chemotherapy with RFA and stenting improved survival (HR = 0.57), suggesting it may optimize treatment efficacy.

Currently, surgical resection with negative margins remains the only potentially curative treatment for HC. However, at the stage of diagnosis, only 20%–30% of patients are suitable for curative resection because of locally advanced malignancies, distant metastases, or poor performance status^[3]^. For most patients with unresectable HC, palliative management aimed at improving quality of life and prolonging survival is the mainstay of treatment^[4,5]^.

The conventional palliative therapies for HC concentrate on alleviating biliary obstruction using endoscopic or percutaneous biliary drainage, which is frequently utilized alongside stent insertion. Both plastic stents (PS) and metal stents (MS) are used for this purpose^[6,7]^. These procedures alleviate symptoms but do not directly inhibit tumor progression. They are also linked to a lot of adverse effects, such as cholangitis and stent occlusion^[5,8]^.

Recently, radiofrequency ablation (RFA) has been recognized as a viable minimally invasive treatment option for several solid tumors, such as hepatocellular carcinoma and colorectal liver metastases^[9]^. This showed that endobiliary RFA could be used to treat malignant biliary strictures. Multiple studies have since evaluated the potential of RFA as a palliative treatment for patients with unresectable HC.

RFA employs a high-frequency alternating current to trigger coagulative necrosis in neoplastic tissue through thermal injury^[9]^. In the context of HC, RFA can be performed endoscopically or percutaneously to achieve local tumor control, maintain biliary patency, and potentially enhance survival rates^[10,11]^. RFA provides benefits including minimally invasive properties, repeatability, and possible synergistic effects when used in conjunction with other treatment techniques such as chemotherapy or radiation therapy^[12]^. Research papers on RFA for HC are growing in number, indicating persistent discussions on the overall efficacy and safety of this technique. Prior systematic reviews and meta-analyses have offered significant insights; yet, they have been restricted by heterogeneous study designs and the incorporation of various tumor types^[13–15]^. There is an absolute need for a new review about the efficacy of RFA for HC patients only. The rapid advancement of RFA techniques and the introduction of new research need an updated and comprehensive assessment of the existing literature.

This systematic review and meta-analysis evaluate the efficacy of RFA in HC, focusing on survival time, stent patency (SP), and safety to help with treatment decisions for such patients.

## Materials and methods

This systematic review and meta-analysis is conducted according to PRISMA guidelines^[16]^ and was registered at PROSPERO with registration No. CRD42024605974. This work has been reported in line with AMSTAR (Assessing the methodological quality of systematic reviews) guidelines and in line with the TITAN criteria^[17,18]^.

## Search strategy

We conducted a comprehensive literature review using the PubMed, Embase, and Scopus databases for studies published from inception to December 2024. The search strategy applied Medical Subject Headings related to HC and RFA (see the Supplemental Digital Content Search Strategy). Additionally, we scrutinized the reference lists of the included studies and pertinent review articles to pinpoint any potentially eligible studies that the electronic search might have missed.

## Inclusion and exclusion

We included articles that reported results of RFA in patients with HC, involved a minimum of 10 patients treated with RFA, and presented data on at least one outcome, including overall survival (OS), SP, or adverse events (AEs), published in English. If the studies included various types of cholangiocarcinoma, we only included those that reported outcomes for HC patients separately. We excluded studies that focused solely on intrahepatic or distal cholangiocarcinoma, those presenting pooled biliary tract cancer outcomes without distinct data for HC, and non-original research, including case reports or conference abstracts.

**What is Already Known**

- Hilar cholangiocarcinoma (HC) is a challenging tumor with limited therapeutic choices, and radiofrequency ablation (RFA) has been investigated as a possible palliative treatment; however, its efficacy and safety remain controversial.

**What This Study Adds**

- This systematic review and meta-analysis suggests that RFA combined with stenting significantly enhances overall survival and stent patency in HC patients compared to stenting alone, while adverse events remain comparable.

**Impact on Research, Practice, or Policy**

- These findings may encourage additional investigation of RFA in HC treatment protocols and impact clinical guidelines, potentially resulting in larger adoption of RFA in palliative care for this patient population.

## Data extraction

Two independent reviewers extracted data from eligible studies using a standardized form. We resolved discrepancies by discussing or consulting with another reviewer. The information extracted from each study includes the first author, year of publication, country, study design, sample size, approach (endoscopic or percutaneous), technical parameters, OS, SP, and AEs. In scenarios where survival data were not directly available, we utilized established methods to extract information from Kaplan–Meier curves or time-to-event analysis.

## Quality assessment

The quality of the included studies was evaluated utilizing the revised Cochrane risk-of-bias tools for randomized controlled trials (RCTs) and non-randomized studies^[19]^. In the assessment of RCTs, we evaluated the following domains: the randomization process, deviations from intended interventions, missing outcome data, outcome measurement, and selection of reported results. We assessed other observational studies based on several criteria: bias from confounding, participant selection, intervention classification, deviations from intended interventions, missing data, outcome measurement, and the selection of reported results. Two reviewers independently conducted the quality assessment, addressing any disagreements through discussion or consultation with a third reviewer.

## Outcomes and definition

Our primary outcome was the pooled hazard ratios (HR) of OS (defined as the survival time of the patients from receiving the therapy to death or the end of the study period). The secondary outcome includes the pooled HR of SP (defined as the time interval between stent placement and stent occlusion or replacement of stent or death), the HR for chemotherapy, and Bismuth types III and IV versus types I and II. We also calculate the odds ratios (ORs) for overall AEs, stent dysfunction, cholangitis, hepatic abscesses, and pancreatitis. Subgroup analyses were performed to investigate potential sources of heterogeneity, including the RFA approach (endoscopic versus percutaneous), stent types (MS versus PS), and study design (RCT versus non-RCTs).

## Statistical analysis

We conducted statistical analyses using the Review Manager (RevMan) software (version 5.4, The Cochrane Collaboration, 2020). For time-to-event outcomes (OS and SP), for studies that did not report these outcomes as HR, we calculated HR using data derived from the Kaplan–Meier curves and time-to-event analysis^[20]^. We calculated the pooled HR using the generic inverse variance method and a random effects model. For dichotomous outcomes, ORs and their 95% confidence intervals (95% CIs) were calculated using a random-effects model with the Mantel–Haenszel techniques. The *I*^2^ statistic was utilized to evaluate heterogeneity across studies, with values categorized as not significant (0%–24%), low (25%–49%), moderate (50%–74%), and high (above 75%)^[21]^. We used funnel plots to evaluate publication bias, following The Cochrane Collaboration’s approach. We conducted sensitivity analyses by sequentially omitting each study to evaluate the reliability of the pooled estimates. A *P* value of less than 0.05 was deemed statistically significant.

## Results

**Figure 1.**
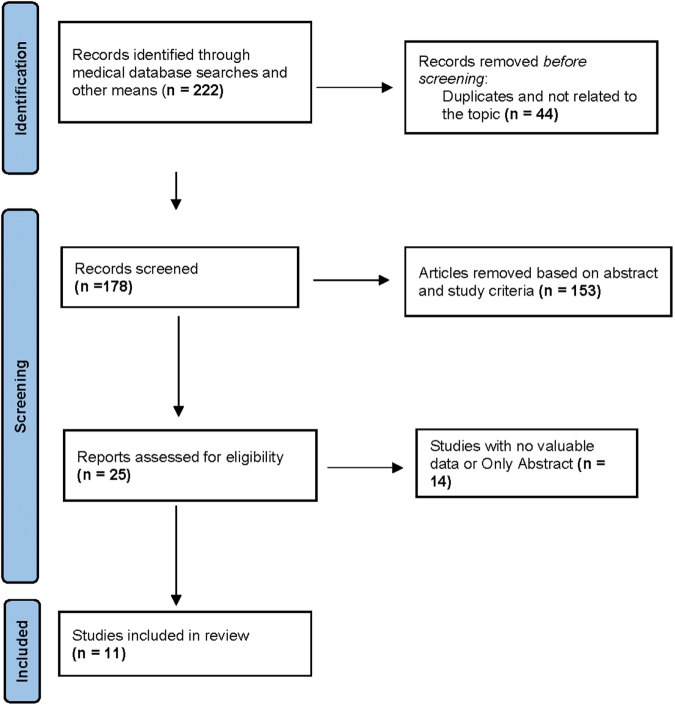
PRISMA flow diagram of study selection.

The database search identified 222 potentially relevant articles for our research. After eliminating 44 duplicates, we selected 178 articles to review their titles and abstracts. We picked 25 articles from this set for a comprehensive analysis of their full texts. Following the application of our inclusion and exclusion criteria, we included 11 studies in our systematic review and meta-analysis^[22–32]^. The study selection process is illustrated in the PRISMA flow diagram (Fig. 1). These eleven included studies comprised seven retrospective cohort studies and four RCTs. The studies were conducted across five countries, with the majority originating from Asia (*n* = 7) and Europe (*n* = 4). The sample sizes ranged from 15 to 256 patients, with a total of 874 patients across all studies. The median follow-up duration ranged from 6 to 48 months. The mean age of patients across studies ranged from 57 to 76 years, with a slight male predominance of 52%–72%. Bismuth-Corlette classification was reported in all the studies, with type III and IV tumors being the most common. Study characteristics and raw outcomes are presented in tables (Tables 1 and 2).

**Table 1:** Characteristics of included studies

Study	Patients	Intervention and RFA setting and probe	Stent type	OS (median/mean) or HR	SPT months or HR	Stent related (occlusion/migration)
Bokemeyer 2019, Germany RS	RFA + S 20	Endoscopic	MS and PS	342 d (±57)	NA	NA
				221 d (±26)		
	S only 22	8–10 W, 90 s, Habib				
Gou 2021, China RS	RFA + S 18	Endoscopic	MS or PS	1.48 (0.87, 2.5)	1.17 (0.68, 2.01)	NA
	S only 17	10 W for 120 s, Habib				
Kang 2021, Korea RCT	RFA + S 15	Endoscopic	MS	230 d (77, 383)	178 d (96.2, 259.8)	6
				144 d (0, 323.1)	122 d (111.2–132.8)	7
	S only 15	7 W for 60–120 s, ELRA				
Gao *et al* 2021, China RCT	RFA + S 25	Endoscopic	PS	0.414 (0.025, 0.762)	NA	NA
	S only 22	7–10 W, 90 s, Habib				
Andrasina 2021, Czech RCT	RFA + S 20	Percutaneous	MS	1.04 (0.55–1.98)	9.6 (0.2, 11.2)	6
					4.5 (0.8, 10.3)	8
	S only 22	10 W,120 s; Habib				
Xia *et al* 2021, China RS	RFA + S 79	Endoscopic	MS and PS	10.2 (8.2–12.8)	NA	NA
				6.0 (4.5–7.5)		
	S only 256	7–10 W, 90 s, Habib				
Oh *et al* 2022, Korea RS	RFA + S 28	Endoscopic	MS	1.024 (0.563–1.864)	1.267 (0.716–2.241)	19
						40
	S only 51	7–10 W, 120 s, ELRA				
Jarosova *et al* 2023, Czech RCT	RFA + S 36	Endoscopic	MS and PS	0.98 (0.68–1.40)	NA	9
						11
	S only 37	8–10 W, 120 s, Habib				
Jagtap *et al* 2024, India RS	RFA + S 23	Endoscopic	MS	1.88 (1.01–3.49)	1.92 (1.20–3.36)	NA
	S only 48	7–10 W, 120 s, ELRA				
Kim *et al* 2024, Korea RS	RFA + S 25	Percutaneous	MS	222 d (143.0–453.0)	188 d (149.0–410.0)	11
				214 d (97.0–268.0)	155 d (60.0–279.0)	20
	S only 31	7 W,120 s, ELRA				
Shin *et al* 2024, Korea RS	RFA + S 32	Endoscopic	MS	337 d (252–404)	242 d (181–309)	24
				296 d (289–383)	168 d (159–281)	27
	S only 32	7 W, 90–120 s, ELRA				

CI, confidence interval; HR, hazard ratio; MS, metal stents; MS, metal stent; N/A, no information available; OS, overall survival; PS, plastic stent; PS, plastic stents; RFA, radiofrequency ablation plus stent; RS, retrospective study; RCT, randomized controlled trial; SPT, stent patency time; S-only, stent only; Habib, Habib EndoHPB, EMcision/Boston Scientific, Mass, USA; ELRA, STARmed Co., Seoul, Korea.

**Table 2:** Characteristics of included studies

Study	Overall adverse events	Cholangitis	Hepatic abscess	Pancreatitis
Kang *et al* 2021	RFA + S = 7	3	0	0
	S only = 12	5	1	1
Oh *et al* 2022	RFA + S = 6	6	NA	NA
	S only = 3	3		
Jarosova *et al*	RFA + S = 6	3	1	1
	S only = 4	3	1	0
Jagtap *et al*	RFA + S = 6	4	NA	1
	S only = 6	3		2
Shin *et al*	RFA + S = 4	3	NA	NA
	S only = 3	2		

N/A, no information available; RFA, radiofrequency ablation plus stent; S-only, stent only.

## Primary outcome

### Survival time

**Figure 2.**
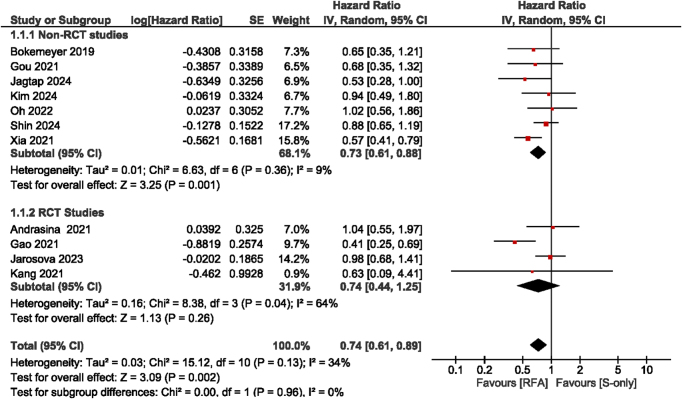
Forest plot of overall survival.

All studies included in the analysis presented data on OS. The results indicated a significant improvement in survival for patients undergoing RFA in comparison to those receiving S-only, evidenced by a pooled HR of 0.74 (95% CI: 0.61–0.89). The studies exhibited low statistical heterogeneity (*I*^2^ = 34%), and the overall effect was statistically significant (*P* = 0.002). In the analysis of subgroups, RCTs indicated no significant difference in survival rates between the RFA and S-only groups (HR: 0.74, 95% CI: 0.44–1.25; *I*^2^ = 64%; *P* = 0.26). Non-RCT studies reveal a significant survival advantage for RFA compared to S-only, with a pooled HR of 0.73 (95% CI: 0.61–0.88; *I*^2^ = 9%; *P* = 0.001) (Fig. 2).

## Secondary outcome

### Stent patency

**Figure 3.**
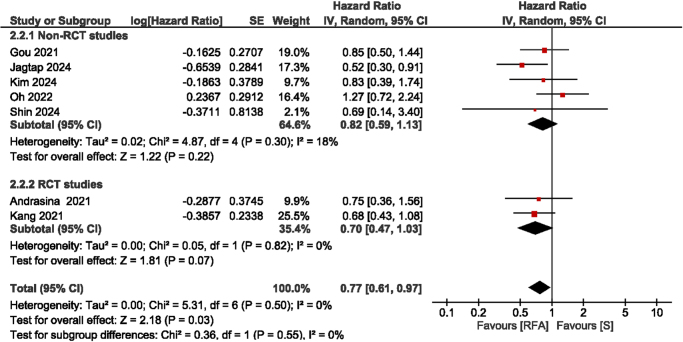
Forest plot of stent patency.

Seven studies provided data regarding SP duration. The combined HR was 0.77 (95% CI: 0.61–0.97; *I*^2^ = 0%; *P* = 0.03), suggesting a statistically significant difference in SP time between the two groups. In the subgroup analysis, both the RCTs and the non-RCT studies revealed no significant difference in SP. The RCT studies reported an HR of 0.70 (95% CI: 0.47–1.03; *P* = 0.07; *I*^2^ = 0%), whereas the non-RCT studies indicated an HR of 0.82 (95% CI: 0.59–1.13; *P* = 0.22; *I*^2^ = 18%) (Fig. 3).

### Stent dysfunction

**Figure 4.**
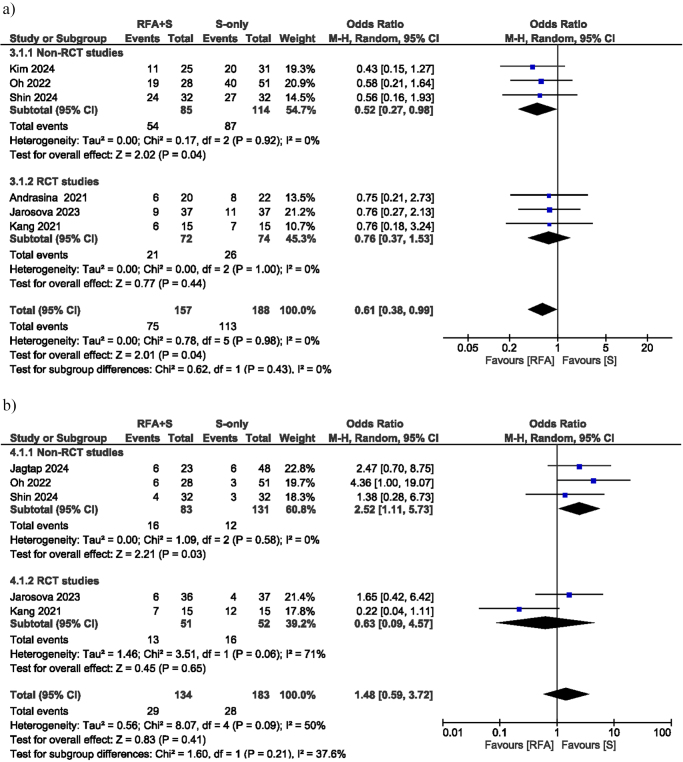
(A) Forest plot of stent dysfunction. (B) Forest plot of overall adverse events.

Six studies reported cases of stent malfunction (occlusion/migration). The OR for stent dysfunction was 0.61 (95% CI: 0.38–0.99; *I*^2^ = 0%; *P* = 0.04), demonstrating a statistically significant difference in the rate of stent dysfunction between the RFA group and the S-only group. The subgroup analysis of RCTs showed that there was no significant difference in the number of stent dysfunction, with an OR of 0.76 (95% CI: 0.37–1.53; *I*^2^ = 0%; *P* = 0.44). In contrast, non-RCT studies exhibited a significant difference, with an OR of 0.52 (95% CI: 0.27–0.98; *I*^2^ = 0%; *P* = 0.04) (Fig. 4a).

### Adverse events

The five studies reported overall AEs, resulting in an OR of 1.48 (95% CI: 0.59–3.72; *I*^2^ = 50%; *P* = 0.41), revealing no significant difference in AEs between the two groups. It was interesting to see that RCT studies didn’t find a difference between the two groups (OR: 0.63, 95% CI: 0.09–4.57; *I*^2^ = 71%, *P* = 0.65), but non-RCT studies did (OR: 2.52, 95% CI: 1.11–5.73, *I*^2^ = 0%, *P* = 0.03) (Fig. 4b). The most frequent AEs were cholangitis (OR: 1.71, 95% CI: 0.78–3.75; *I*^2^ = 13%; *P* = 0.18), pancreatitis (OR: 1.03, 95% CI: 0.19–5.50; *I*^2^ = 0%; *P* = 0.98), and liver abscess (OR: 0.62, 95% CI: 0.07–5.24; *I*^2^ = 0%; *P* = 0.66), all of which demonstrated no significant differences between the two cohorts (Supplemental Digital Content Figures 1–3, available at: http://links.lww.com/JS9/E976).

### Chemotherapy

**Figure 5.**
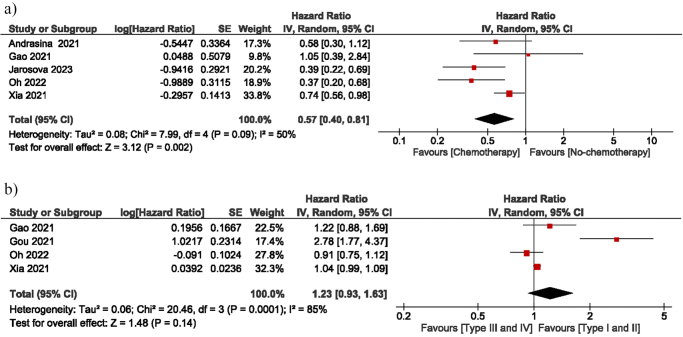
(A) Forest plot of chemotherapy. (B) Forest plot of survival regarding bismuth type II vs. type III.

Five studies evaluated the impact of chemotherapy on patient OS. The pooled HR was 0.57 (95% CI: 0.40–0.81; *I*^2^ = 50%; *P* = 0.002), suggesting a significant difference in OS between patients undergoing chemotherapy and those who are not (Fig. 5a).

### Bismuth types I and II vs types III and IV

Only four studies provided data on the OS differences between Bismuth types I and II and types III and IV HC. The pooled HR was 1.23 (95% CI: 0.93–1.63; *I*^2^ = 85%; *P* = 0.14), suggesting no significant difference in survival between the two groups (Fig. 5b).

### MS vs PS

The HR for survival with the use of both MS and PS was 0.71 (95% CI: 0.53–0.95; *I*^2^ = 37%; *P* = 0.02). The HR for MS alone was 0.87 (95% CI: 0.70–1.07; *I*^2^ = 0%; *P* = 0.19), suggesting no significant difference in survival when compared to the combination of MS and PS. Conversely, PS alone demonstrated a significant survival difference, with an HR of 0.41 (95% CI: 0.25–0.69; *P* = 0.0006) (Supplemental Digital Content Figure 4, available at: http://links.lww.com/JS9/E976).

### Quality assessment and publication bias

The studies included in the analysis were generally assessed as having moderate quality. In the RCTs assessed, two revealed a low risk of bias, whereas the remaining two showed a moderate risk of bias, as determined by the RoB 2 tool. All seven non-RCT studies showed a moderate risk of bias, which was evaluated by the ROBINS-1 tool (Supplemental Digital Content Figures 8–9, available at: http://links.lww.com/JS9/E976).

The visual analysis of the funnel plots suggested no evidence of publication bias regarding all outcomes. Sensitivity analysis indicated that no individual study significantly influenced the outcome (Supplemental Digital Content Figure 5–7, available at: http://links.lww.com/JS9/E976).

## Discussion

This systematic review and meta-analysis, comprising 11 studies and 874 patients, provides a comprehensive evaluation of the efficacy and safety of RFA in the treatment of HC. The findings suggest that RFA + S, offers significant survival benefits and improved biliary drainage compared to S-only, making it a promising palliative intervention for patients with unresectable HC. The pooled HR for OS was 0.74 (95% CI: 0.61–0.89), indicating a 26% reduction in the risk of death for patients undergoing RFA. This survival advantage was consistent across most studies, with low heterogeneity (*I*^2^ = 34%), underscoring the reliability of these findings.

Given the poor prognosis of HC, the significant improvement in survival associated with RFA is particularly noteworthy. Palliative therapies focused on symptom relief and survival extension constitute the primary treatment approach for most patients^[14]^. Conventional methods, including biliary stenting, relieve obstruction yet fail to tackle tumor progression^[4]^. RFA induces coagulative necrosis in tumor tissue, providing a mechanism for local tumor growth control and potentially prolonging survival^[12]^. Several RCTs have proven that RFA + S is more effective than the S-only approach for malignant biliary obstruction^[24,33]^. A recent RCT revealed that both the RFA + S and S-only groups exhibited comparable outcomes for biliary strictures^[27]^. Previous meta-analyses included studies including various types of strictures and evaluated diverse outcomes^[12,15]^. This meta-analysis includes patients with HC and evaluates the efficacy of RFA for this population. The survival advantage of RFA was more significant in non-RCTs (HR = 0.73, *P* = 0.001) than in RCTs (HR = 0.74, *P* = 0.26). This discrepancy may indicate variations in patient selection, as non-RCTs could include a wider spectrum of patients, including those with more advanced diseases. The smaller sample sizes and stricter inclusion criteria in RCTs could limit their capacity to identify significant differences. Furthermore, RCTs follow critical guidelines and protocols, while non-RCTs are just retrospective analyses of the available data.

RFA revealed a significant improvement in SP, with a pooled HR of 0.77 (95% CI: 0.61–0.97). This indicates that RFA prolongs biliary drainage, thereby decreasing the necessity for stent replacement or reintervention. The mechanism likely includes tumor debulking, which inhibits tumor ingrowth or overgrowth that may result in stent occlusion. The OR for stent dysfunction (occlusion or migration) was 0.61 (95% CI: 0.38–0.99), reflecting a 39% decrease in the risk of stent-related complications associated with RFA. This finding is significant, as stent dysfunction is common in HC management, often necessitating further interventions and elevating patient morbidity. This is also reported by most studies that RFA not only improves survival but also prolongs SP.

The safety of RFA is a crucial factor, particularly in palliative care, where the reduction of AEs is essential^[34]^. The pooled analysis indicated no significant difference in overall AEs between the RFA + S and S-only groups (OR = 1.48, *P* = 0.41). Specific AEs, including cholangitis (OR = 1.71, *P* = 0.18), pancreatitis (OR = 1.03, *P* = 0.98), and liver abscess (OR = 0.62, *P* = 0.66), revealed no significant differences. Non-RCT studies indicated a higher incidence of AEs in the RFA group (OR = 2.52, *P* = 0.03), possibly indicating variation in patient selection and procedural expertise in real-world settings. This also could be attributed to differences in procedural expertise, patient characteristics, or underreporting in prospective trials. Previous studies also have shown similar results^[12,35]^. A recent study reported that patients with type 2 diabetes mellitus, segmental RFA, and postoperative single stent drainage may be risk factors for such AEs^[36]^. These findings underscore the necessity of conducting RFA in specialized centers that have the expertise to deal with potential AEs.

Our analysis revealed no significant difference in survival between Bismuth type I/II and type III/IV tumors (HR = 1.23, *P* = 0.14), indicating that tumor classification may not be a key factor influencing outcomes in this context. The type of stent selected significantly affected survival outcomes. The application of PSs alone demonstrated a notable survival advantage (HR = 0.41, *P* = 0.0006), while MSs alone exhibited no significant difference (HR = 0.87, *P* = 0.19). This finding may indicate variations in SP, complication rates, or patient selection, necessitating further investigation.

Chemotherapy has emerged as a critical factor in improving survival for patients with HC, evidenced by a pooled HR of 0.57 (95% CI: 0.40–0.81, *P* = 0.002) for patients undergoing chemotherapy versus those who are not. This finding is consistent with existing clinical guidelines that advocate for chemotherapy as the primary systemic treatment for unresectable HC. The integration of RFA with chemotherapy indicates synergistic advantages, as RFA facilitates local tumor management while chemotherapy targets systemic disease dissemination. An RCT comparing patients treated with RFA plus chemotherapy to those receiving RFA alone provides evidence that the combination therapy significantly enhances both survival rates and SP^[37]^. The findings underscore the potential benefits of combining RFA with systemic therapies to improve outcomes for patients with advanced HC^[38]^. Future research should focus on identifying the optimal sequencing and timing of these therapies. Investigating the timing of RFA administration with chemotherapy – before, during, or after – could enhance treatment strategies and optimize patient outcomes. These studies are crucial for advancing a personalized and effective strategy for managing this complex malignancy. Combining local and systemic therapies enables clinicians to provide patients with extended survival and enhanced quality of life, representing a promising approach for advancing healthcare treatment.

Although this meta-analysis provides significant insights, it is important to acknowledge the limitations. Most of the studies included were retrospective (*n* = 7), which may introduce bias and confounding factors. There are some differences in RCTs and non-RCTs results that should be kept in mind while interpreting these results. The variability in study designs, patient populations, and RFA techniques may influence the generalizability of the findings. Some studies did not report all outcomes specifically for HC patients; however, we utilized the data as a whole, which should also be considered. The HR of the studies was calculated from Kaplan–Meier curves for time-to-event outcomes or log-rank tests, which may have affected the outcomes, especially for the SP. Despite our efforts to assess publication bias, the possibility of unpublished negative results cannot be entirely ruled out. Additionally, the relatively short follow-up periods in most studies limit our understanding of the long-term outcomes and potential late AEs of RFA in HC patients. Other conditions, including the absence of standardized criteria for RFA eligibility, especially concerning tumor size, vascular proximity, and anatomical subtype, may have some impact on the outcomes of these studies.

Future research should prioritize prospective RCTs comparing RFA to S-only treatments, alongside investigations into the optimal integration of RFA with systemic therapies. Research on biomarkers or imaging features that forecast responses to RFA may enhance patient selection. Additionally, long-term follow-up studies are essential to assess the durability of RFA outcomes and detect any late complications. Comparative cost-effectiveness analyses of RFA and alternative palliative treatments would yield important insights for healthcare resource allocation.

## Conclusion

This meta-analysis demonstrates that RFA + S is a promising palliative treatment for unresectable HC, offering significant survival benefits and improved SP, without significantly increasing AEs.

## Data Availability

The supporting data of this study are available in supplementary materials and more information can be found on request from the corresponding author (YJF), upon reasonable request.

## Research registration unique identifying number (UIN)

This systematic review and meta-analysis was registered at PROSPERO (https://www.crd.york.ac.uk/PROSPERO/) with registration number CRD42024605974.
